# [Retracted] Selenium-binding protein 1 may decrease gastric cellular proliferation and migration

**DOI:** 10.3892/ijo.2025.5731

**Published:** 2025-02-17

**Authors:** Chenjing Zhang, Wen Xu, Wensheng Pan, Nana Wang, Guogang Li, Xiaoyuan Fan, Xiang Xu, Shengrong Shen, Undurti N. Das

Int J Oncol 42: 1620-1629, 2013; DOI: 10.3892/ijo.2013.1850

Following the publication of this paper, it was drawn to the Editor's attention by a concerned reader that, for the flow cytometric plots shown in Fig. 9A on p. 1628, a pair of data panels showed a surprisingly high level of partially overlapping data, given that the results from differently performed experiments were intended to have been shown in these figure parts. Upon performing an independent analysis of the data in this paper, a series of further issues concerning the data were noted; first in Fig. 9B, a separate pair of flow cytometric plots appeared to have been duplicated in their entireties. In addition, two pairs of data panels in Fig. 6A and B (showing the senescence of gastric cancer cells) contained overlapping sections of data; the control panels for the migration assay data in Fig. 7A and B were overlapping, albeit the orientation of the overlapping data sections was different.

Owing to the large number of duplications of data, and other potential anomalies, that were identified in this paper, the Editor of *International Journal of Oncology* has decided that it should be retracted from the Journal on account of a lack of confidence in the presented data. The authors were asked for an explanation to account for these concerns, but the Editorial Office did not receive a satisfactory reply. The Editor apologizes to the readership for any inconvenience caused.

